# Draft Whole-Genome Sequence of *Penicillium simplicissimum* A4, a Putative Endophyte from *Echium plantagineum*

**DOI:** 10.1128/mra.00854-22

**Published:** 2022-10-26

**Authors:** Stacey Fisher, Dewald De Villers, Morne Du Plessis, Kacey Hattingh, Crishe Saulse, Gerhard Basson, Adelé Barker, Augustine Innalegwu Daniel, Ali Al-Hashimi, Arina Hitzeroth, Thulani Makhalanyane, Vuyo Mavumengwana, Arun Gokul, Marshall Keyster, Ashwil Klein

**Affiliations:** a Plant Omics Laboratory, Department of Biotechnology, University of the Western Cape, Bellville, South Africa; b Environmental Biotechnology Laboratory, Department of Biotechnology, University of the Western Cape, Bellville, South Africa; c Sequencing Core Facility, National Institute for Communicable Diseases, Johannesburg, South Africa; d Plant Biotechnology Research Group, Department of Biotechnology, University of the Western Cape, Bellville, Western Cape, South Africa; e Institute for Microbial Biotechnology and Metagenomics, University of the Western Cape, Bellville, Western Cape, South Africa; f Department of Biochemistry, Genetics and Microbiology, University of Pretoria, Pretoria, South Africa; g Centre for Tuberculosis Research, South African Medical Research Council, Department of Biotechnology, University of the Western Cape, Bellville, Western Cape, South Africa; h Department of Plant Sciences, University of the Free State, Phuthadithjaba, South Africa; University of Maryland School of Medicine

## Abstract

We report the draft whole-genome sequence of the putative endophytic fungus Penicillium simplicissimum A4, isolated from the roots of Echium plantagineum plants. The genome was sequenced using PacBio technology with an estimated genome size of 39 Mb.

## ANNOUNCEMENT

Penicillium simplicissimum is an endophytic fungus belonging to the Aspergillaceae family (1, 2). Members of the Aspergillaceae family are widely distributed and have been isolated from diverse habitats including soil, vegetation, and food products (3–5).

Echium plantagineum plants were collected from Durbanville, South Africa (33°5147.5S, 18°3917.2E), in March 2019. Root material was surface sterilized with 70% ethanol for 3 min, 2.5% sodium perchlorate for 5 min, and 70% ethanol for 1 min and washed 5 times with reverse osmosis (RO) water (6). Sterilized roots were homogenized in a 0.9% saline (NaCl) solution using a sterile mortar and pestle. The homogenate was diluted (1:100), plated onto potato dextrose agar (PDA) containing 100 μg/mL chloramphenicol, and incubated at 25°C for 7 days. A single colony was picked, restreaked onto PDA, and incubated at 25°C for 7 days. Approximately 100 mg of biomass was collected, and total genomic DNA was extracted using the Zymo Research Quick-DNA Fungal-Bacterial Miniprep kit (Zymo Research, catalog number D6005). The purity and concentration of the DNA were measured using a NanoDrop 2000 spectrophotometer (Thermo Scientific). The isolate A4 was identified as *Penicillium simplicissimum* (99% identity to accession KY315584.1) based on the DNA sequence of the internal transcribed spacer (ITS) region (ITS1 and ITS4), which has been deposited in GenBank under the accession no. OP512539.

Genomic DNA sequencing was performed at Inqaba Biotechnical Industries (Pretoria, South Africa) with the single-molecule real-time (SMRT) technology from PacBio. The genomic DNA (~1 μg) was size selected using the AMPure PB bead size selection kit (Pacific Biosciences, Menlo Park, CA). The HiFi SMRTbell library was constructed using the SMRTbell template preparation kit v3.0 and SMRTlink software v11 according to the manufacturer’s instructions. The HiFi SMRTbell library was sequenced at Inqaba Biotechnical Industries (Pty) Ltd. (Pretoria, South Africa) using the PacBio Sequel IIe system in circular consensus sequencing (CCS) mode with 110× coverage per the manufacturer’s instructions, resulting in a total read length of 531,767 bp and an *N*_50_ read of 5,000 kb.

The primary genome assembly was performed with Canu v2.2 (7) with default parameters and a genome size setting at 35 Mb. The assembly resulted in a total genome size of 39 Mb with 50.61% GC content, 110× sequencing coverage, 196 contigs, *N*_50_ contig length of 3,965.722 kb, and a maximum contig size of 6,478,589 bp. The genome assembly was validated using BUSCO v. 5.4.2 (8), with default parameters, which showed a 96% hit against 4,191 fungal genes. Gene prediction was performed using Augustus 3.4.0 (9), with default parameters against Aspergillus nidulans as reference, and produced 11,862 protein-coding genes.

### Data availability.

The assembled genome sequence of *Penicillium simplicissimum* isolate A4 is available at GenBank under the accession number JAMZTW000000000 with BioProject number PRJNA855613, BioSample number SAMN29498895, and SRA accession number SRR20281321.
